# Camp NERF: methods of a theory-based nutrition education recreation and fitness program aimed at preventing unhealthy weight gain in underserved elementary children during summer months

**DOI:** 10.1186/s12889-016-3765-7

**Published:** 2016-10-26

**Authors:** Laura C. Hopkins, Mary Fristad, Jacqueline D. Goodway, Ihuoma Eneli, Chris Holloman, Julie A. Kennel, Bernadette Melnyk, Carolyn Gunther

**Affiliations:** 1 Department of Human Sciences, Human Nutrition Program, The Ohio State University, 262B Campbell Hall, 1787 Neil Avenue, Columbus, OH 43210 USA; 2 Department of Psychiatry and Behavioral Health, The Ohio State University Wexner Medical Center, 1670 Upham Drive Suite 460G, Columbus, OH 43210-1250 USA; 3 Department of Human Sciences, Kinesiology Program, College of Education & Human Ecology, The Ohio State University, A244 305 Annie & John Glenn Ave, Columbus, OH 43210 USA; 4 Nationwide Children’s Hospital; The Ohio State University, 700 Children’s Drive, Columbus, OH 43205 USA; 5 Department of Statistics, The Ohio State University, 404 Cockins Hall, 1958 Neil Ave, Columbus, OH 43210 USA; 6 Department of Human Sciences, Human, The Ohio State University, 315 Campbell Hall, 1787 Neil Avenue, Columbus, OH 43210 USA; 7 College of Nursing, The Ohio State University, 1585 Neil Avenue, Rm. 145, Columbus, OH 43210 USA; 8 Department of Human Sciences, Human Nutrition Program, The Ohio State University, 313 Campbell Hall, 1787 Neil Avenue, Columbus, OH 43210 USA

**Keywords:** Nutrition, Physical activity, Elementary children, Behavioral intervention, Childhood obesity prevention

## Abstract

**Background:**

The number of obese children in the US remains high, which is problematic due to the mental, physical, and academic effects of obesity on child health. Data indicate that school-age children, particularly underserved children, experience unhealthy gains in BMI at a rate nearly twice as fast during the summer months. Few efforts have been directed at implementing evidence-based programming to prevent excess weight gain during the summer recess.

**Methods:**

Camp NERF is an 8-week, multi-component (nutrition, physical activity, and mental health), theory-based program for underserved school-age children in grades Kindergarten - 5th coupled with the USDA Summer Food Service Program. Twelve eligible elementary school sites will be randomized to one of the three programming groups: 1) Active Control (non-nutrition, physical activity, or mental health); 2) Standard Care (nutrition and physical activity); or 3) Enhanced Care (nutrition, physical activity, and mental health) programming. Anthropometric, behavioral, and psychosocial data will be collected from child-caregiver dyads pre- and post-intervention. Site-specific characteristics and process evaluation measures will also be collected.

**Discussion:**

This is the first, evidence-based intervention to address the issue of weight gain during the summer months among underserved, school-aged children. Results from this study will provide researchers, practitioners, and public health professionals with insight on evidence-based programming to aid in childhood obesity prevention during this particular window of risk.

**Trial Registration:**

NCT02908230/09-19-2016

## Background

While recent reports indicate a plateau in the rate of childhood obesity in the United States, the number of obese children remains high [1]. In 2011–2012, obesity affected 17 % of US youth, with 31.8 % being classified as overweight or obese [1]. Significant differences in prevalence of obesity exist between racial, ethnic and age groups. Non-Hispanic black and Hispanic youth are significantly more affected by obesity than their non-Hispanic White and non-Hispanic Asian peers [1]. Additionally, there appears to be a developmental trajectory in prevalence of obesity as 8 % of 2- to 5-year olds, 17.7 % of 6- to 11-year olds, and 20.5 % of 12- to 19-year olds were classified as obese in 2011–2012 [1]. So while it appears that the rise in obesity has tapered off, the prevalence of overweight and obesity among US youth remains concerning due to its devastating consequences, which affect the physical and mental health of children, as well as their academic success [2, 3].

Emerging research has begun to point to particular windows of risk for child weight gain. Troubling data indicate that school-age children experience unhealthy gains in BMI at a rate nearly twice as fast during the summer months when school is out of session compared to the school year [4–10]. African American and Hispanic, minority groups and economically disadvantaged children, subpopulations already at increased risk for obesity, as well as girls, may be particularly vulnerable to unhealthy weight gain during these non-academic months [1]. Limited knowledge of the external factors that lead to altered diet and physical activity during the summer time is available to adequately explain the unfavorable weight gain occurring in many children during this window of risk [11].

The rise and current status of obesity in the US has occurred at such a rapid rate that it cannot solely be attributed to biological changes [12]. While obesity rates have been rising over the past several decades, the US food environment has also been changing drastically, providing convenient access to an abundance of inexpensive, highly palatable, energy-dense foods [13]. Thus, the current prevalence of childhood obesity and demonstrated increase in obesity during the summer months could be a response to children’s increased exposure to the food environment [13], which they have less frequent access to during the school year, and lack of structured physical activity. Schools play a critical role in promoting healthy diet and physical activity behaviors during the academic year [14]. During the summer months, however, children lose access to this structured environment (e.g., provision of healthy snacks and meals; opportunity for structured and unstructured physical activity; nutrition, physical activity, and health related policies and programs).

The United States Department of Agriculture (USDA) aims to provide access to healthy, nutritious meals to children during the summer months through the USDA Summer Food Service Program (SFSP) [15]. Unfortunately, attendance at USDA SFSP sites, especially open sites, and amount of meals served tends to be low. According to the Food Research Action Center, only 15.8 % of free or reduced-cost school lunch participants received lunch in the summer of 2015 nationally [16]. In Ohio, only 10.8 % of free or reduced-cost school lunch participants participated in the SFSP [17]. Several stakeholders have hypothesized that these low numbers are due to a lack of structured programming at the sites to attract children. Few efforts have been directed at designing evidence-based nutrition and physical activity programs to equip underserved children with the necessary knowledge, skills, and other resources to prevent excess weight gain during the summer recess.

Camp Nutrition Education Recreation and Fitness (NERF) is a multi-component, evidence-based nutrition, physical activity, and mental health intervention that is coupled with USDA SFSP open sites in Columbus, Ohio. To our knowledge, this is the first multi-component, evidence-based intervention to address the disproportionate childhood weight gain in underserved children during the summer months. The long-term goal is to develop and implement effective theory-based community nutrition and physical activity interventions for childhood obesity prevention, particularly in underserved minority children, aimed at empowering children to make healthy dietary and physical activity choices and achieve a healthy weight and, ultimately, overall optimal health and wellness. The primary aims of this research project are to:

1. Evaluate the efficacy of Camp NERF to improve child nutrition, physical activity, mental health, and anthropometric outcomes.
  - Hypothesis 1.1: Diet quality, physical activity and sedentary time, positive and negative affect, BMI z-scores, and waist circumference (WC) z-scores will improve more from baseline to post-intervention among children participating at the Enhanced Care sites compared to Standard Care and Active Control sites.
2. Evaluate the efficacy of Camp NERF to improve caregiver self-efficacy for establishing healthy family nutrition and physical activity practices, amount of physical activity, and BMI.
  - Hypothesis 2.1: Caregiver self-efficacy scores for establishing healthy family nutrition and physical activity practices, physical activity score, and BMI will improve more from baseline to post-intervention among families participating at the Enhanced Care sites compared to the Standard Care and Active Control sites.
3. Evaluate the efficacy of Camp NERF to improve youth mentor nutrition, physical activity, and anthropometric outcomes.
  - Hypothesis 3.1: Diet quality, physical activity and sedentary time, positive and negative affect, BMI z-scores, and waist circumference (WC) z-scores will improve among youth mentors from baseline to post-intervention.

## Methods

### Camp NERF theoretical framework

Commonalities among the relatively few successful community-based childhood obesity prevention efforts include: theoretical framework to the intervention, multi-component strategies, direct or indirect engagement of caregivers, and specific behavioral targets [18]. It has become widely accepted that use of a theoretical framework in the design of behavior change interventions is an essential ingredient for achieving positive outcomes. The Camp NERF intervention is guided by the social ecological model (SEM) and social cognitive theory (SCT) [19–21].

According to the socio-ecological framework, there is a complex interplay of factors at multiple levels of influence that determine a child’s weight status, health, and wellness [22]. A child’s risk for obesity is influenced by personal factors, such as genetics and diet and physical activity behaviors. These personal factors are, in turn, influenced by multiple external layers of influence including caregiver/family practices and behaviors, environmental settings (home, school, community), various organizational sectors (education, government, public health, leisure, recreation), and social norms and values (societal rules that guide attitudes, beliefs, and behaviors, peer influence). The socio-ecological framework provides a theory-based approach to investigating the problem of childhood obesity and an understanding of the deep complexity of the etiology of this disease. It also serves as a useful tool in the design of theory-based behavior change interventions – and underlines the necessity in conducting cross-disciplinary research to effectively diminish the problem of childhood obesity.

The SCT broadly used among community nutrition researchers, proposes that behavior change results from a reciprocal relationship between personal and external factors [20]. An individual needs the personal resources to enact the desired behavior, which includes: knowledge and skills (ability to perform desired behavior); cognitive behavior techniques (goal-setting, problem solving, coping strategies); and self-efficacy (confidence in one’s ability to enact the behavior). Regarding cognitive behavioral techniques, self-control is achieved via goal-setting. When goals are not achieved, alternative skills, such as problem solving and coping strategies can be employed to attain initial goals or set new, more achievable goals [20]. Also worth noting, children and adolescents who develop proficiency in general cognitive behavior techniques experience a sense of personal empowerment. In turn, this alleviates the mental health symptoms associated with overweight and obesity (poor self-concept and symptoms of anxiety and depression), leading to subsequent diet and physical activity related behavior change [23]. Cognitive behavior techniques are either vastly underdeveloped or missing from the curriculum of most childhood obesity prevention interventions [24, 25]. The Camp NERF intervention utilizes two evidence-based curricula that incorporate cognitive behavioral techniques - Coordinated Approaches to Child Health (CATCH) [26] and Creating Opportunities for Personal Empowerment (COPE) [27]. Additionally, goal-setting opportunities strategies are integrated into the Camp NERF curriculum and achievement of goals are tracked with goal-setting necklaces.

Under the SCT, environmental or external factors also play a part in determining behavior. With regards to child diet and physical activity behaviors, examples include: role modeling (caregivers, teachers, peers), availability of healthy meals and snacks, opportunity for indoor or outdoor physical activity and structure provided by daily routines [22]. Due to the complexity of behavior origination and change, the most efficacious interventions have been multi-component in design and included either direct or indirect caregiver engagement. As the nutritional gatekeepers of the household, caregivers play a major role in shaping the eating behaviors of their children and thus must be included as an intervention target [28]. Caregivers help children establish and reinforce target behaviors by role modeling intake of healthy foods, setting expectations for healthy food intake, and making healthy foods available [29]. The same principle holds true for physical activity related behaviors – that is, caregivers heavily influence their child(ren)’s engagement in physical activity and exercise, as well as sedentary and screen time [30]. Thus, caregivers must be involved either directly or indirectly in behavioral interventions directed at the child. The food and physical activity environments, caregivers, and peers are all targets of the Camp NERF intervention.

**Fig. 1:**
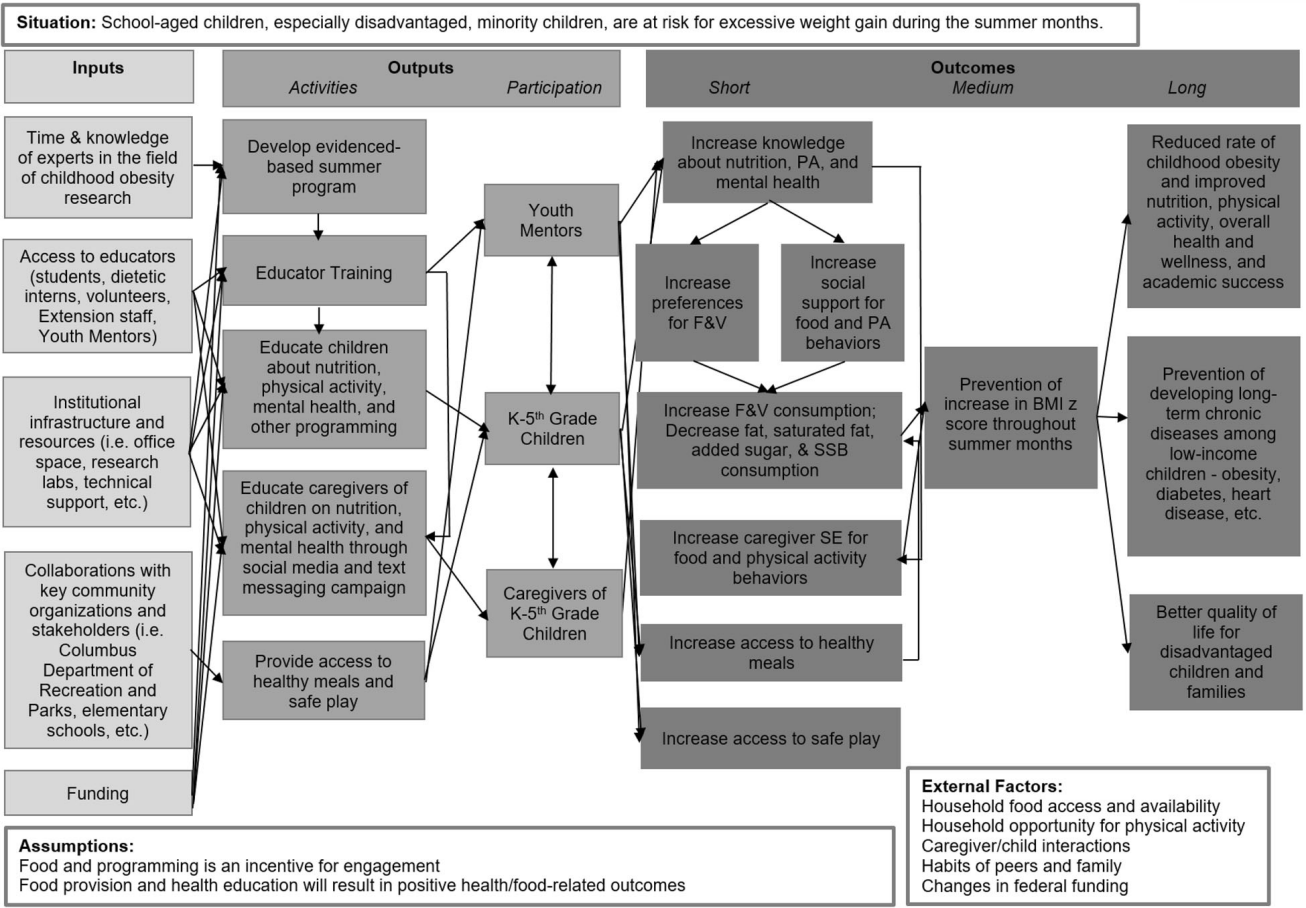
Camp NERF 2015 Theoretical Framework

In sum, due to the inherent complexity of behavior, use of theoretical frameworks, such as the SEM and SCT, are absolutely critical in the design and conduct of behavioral interventions. Please refer to Fig. 1 for the Camp NERF theoretical framework.

### Research design

**Fig. 2:**
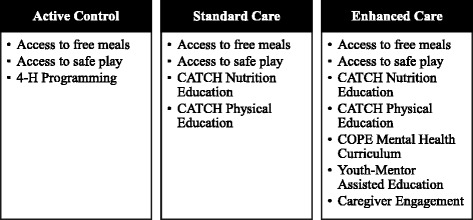
Overview of the Camp NERF Treatments

Camp NERF is an 8-week pre-test, post-test group site-randomized controlled trial. It is a multi-component nutrition, physical activity, and mental health education intervention coupled with the USDA SFSP, specifically open sites located at public elementary schools. Through daily access to healthy foods, safe play and structured physical activity, along with engagement in an evidence-based health behavior educational curriculum, Camp NERF is designed specifically to prevent unintended, unhealthy weight gain during the summer months in underserved school-aged children. Potential sites were identified by a community partner whose responsibility it is to support SFSP sites in Franklin County, OH, and were considered eligible if they were: 1) an elementary school; 2) a USDA SFSP open site; and 3) lacking structured programming. Twelve sites were identified as meeting these inclusion criteria and will be randomized to one of three treatment or programming groups: 1) Enhanced Care (nutrition, physical activity, and mental health programming); 2) Standard Care (nutrition and physical activity programming); and 3) Active Control (non-nutrition, physical activity, and mental 4H programming). Figure 2 provides an overview of the three treatments for Camp NERF.

Power was calculated using change in BMI z-score as the outcome of interest. Based on the results from previous pilot test [31, 32], we assume that between-subject variation is normally distributed with a standard deviation of 1.03 and that between-site variation (nested within treatment group) is negligible. Under these assumptions, recruiting 20 subjects per site (planning for 20 % attrition) provides approximately 70 % power to detect a difference of 0.5 points in change in BMI between the treatment group and either of the two control groups using a one-sided test at alpha = 0.05. The model used is described further in the Data Analysis section.

USDA SFSP open feeding sites operate 5 days a week from mid-June through the beginning of August. Their hours of operation will be approximately 9:00 AM – 3:00 PM, although these times vary slightly by site. Camp NERF program (Enhanced Care, Standard Care, or Active Control) will occur 2 days per week for approximately 4 hours each day. This is expected to equate to 64 h of direct exposure per child, a sufficient dose for eliciting behavior change in the targeted outcomes [33–42]. Table 1 provides an overview of a sample day at an Enhance Care site.

**Table 1:** Camp NERF daily curricula delivery schedule at enhanced care sites

Time	Grades K-2	Time	Grades 3–5
8:30 AM	Arrival; Engage with participants before and during breakfast	8:30 AM	Arrival; Engage with participants before during breakfast
10:00 AM	Nutrition Education	10:00 AM	Physical Education
10:00 AM	Discussion	10:00 AM	*Warm-up*
10:10 AM	*Learning Activity*	10:05 AM	*Go Fitness*
10:25 AM	*Physical Activity*	10:20 AM	*Go Activity*
		10:40 AM	*Cool-down*
10:45 AM	Mental Health	10:45 AM	Mental Health
11:15 AM	Physical Education	11:15 AM	Nutrition Education
11:15 AM	*Warm-up*	11:15 AM	Discussion
11:20 AM	*Go Fitness*	11:25 AM	*Learning Activity*
11:35 AM	*Go Activity*	11:40 AM	*Physical Activity*
11:55 AM	*Cool-down*		
12:00 PM	Lunch; Lunchtime Engagement and Trivia	12:00 PM	Lunch; Lunchtime Engagement and Trivia

### Participants and recruitment

The target population for Camp NERF is underserved minority children entering kindergarten through fifth grade and their adult caregiver from urban neighborhoods in Columbus, Ohio. Participants will be recruited through a variety of methods including, but not limited to, school announcements, emails, flyers, phone calls, and neighborhood canvassing. Prior to enrollment in the study, a consent form, parental permission form, and assent form will be completed by the caregiver and child, respectively.

Also, data will be collected from high school students who will serve as Youth Mentors for the child participants at the Camp NERF Enhanced Care sites. The Youth Mentors will be recruited through a collaborating partner, the Godman Guild Association that provides internships to high-school aged students during the summer months through Temporary Assistance for Needy Families (TANF) funding. Prior to study enrollment, a parental permission form and assent form will be completed by the caregiver and Youth Mentor, respectively, for those under the age of 18. Youth Mentors 18 years of age or older will complete consent forms prior to enrollment in the study. Individuals who are not interested in enrolling in the study will still be able to participate as Camp NERF Youth Mentors.

### Data collection

#### Data collection training

Data collectors will consist of undergraduate and graduate students from nutrition, public health or other related fields, as well as registered dietitian nutritionists. All data collectors will undergo an 8-hour data collection training, which will include didactic sessions followed by role-playing to practice techniques and become familiar with the instruments. At the end of the 8-week intervention prior to post-test data collection, data collectors will complete a 2-hour review training to reacquaint themselves with the instruments and learn additional post-test data collection feedback surveys.

#### Impact measures

Child-caregiver dyads and youth mentors will be interviewed at baseline and post-intervention using the Camp NERF Child Assessment Form, Camp NERF Adult Assessment Form, and Camp NERF Youth Mentor Assessment Form, respectively. Each assessment form consists of validated nutrition, physical activity, and mental health questionnaires. Three (2 weekdays and 1 weekend day) 24-hour dietary recalls will be conducted for both the children and youth mentors. Heights, weights, blood pressure, and waist circumference measurements will be taken for the children and youth mentors. Caregivers will self-report height and weight. Table 2 provides the Camp NERF Evaluation Chart and outlines all validated surveys and outcomes measured for participants.

**Table 2:** Camp NERF evaluation chart for children, caregivers, and youth mentors

Outcomes	Goal	Measure	b0	T1
Child Outcomes				
Nutrition				
Food Attitudes and Preferences	Increase preference for fruits and vegetables	Fruit and Vegetable Preferences *Domel 1993* ^85^	X	X
Dietary Intake	Increase quality of diet (increase fruit and vegetable intake (quantity and variety), decrease intake of foods high in solid fats and added sugars; decrease sugar-sweetened beverages)	Caregiver-assisted 24-hour diet recall *Burrows et al 2010* ^86^ *Baxter et al 2003* ^87^ *Baxter et al 2009* ^88^	X	X
Physical Activity				
Physical activity level and sedentary time	Increase estimated active time and decrease estimated time in sedentary behavior	3rd-5th^:^ SPAN questionnaire *Hoelscher et al 2010* ^89^	X	X
Mental Health				
Self-concept	Increase positive affect	PANAS Survey *Laurent et al 1999* ^90^	X	X
	Decrease negative affect			
	Retain positive changes in self-concept			
Social Support				
Social Support for Food and Physical Activity Habits	Increase social support for food and physical activity habits	Social Support Scale for Food and Physical Activity Habits	X	X
		*Gadhoke 2015* ^91^		
Social Support for Healthy and Unhealthy Eating	Increase social support for healthy eating and decrease social support for unhealthy eating	Social Support Scale for Healthy and Unhealthy Eating *Fitzgerlad 2013* ^92^	X	X
Biometrics				
Height and Weight	Prevent unhealthy weight gain	Hopkins Road Rod Portable Stadiometer (Height) BalanceFrom High Accuracy Digital Scale (Weight) 2000 CDC sex-specific BMI-for-age growth chart^93^	X	X
Waist Circumference	Prevent increase in waist circumference	MyoTape tape measure CDC Waist Circumference Tables ^94^	X	X
Blood Pressure	Prevent increase in blood pressure	Panasonic Portable Blood Pressure Monitor NHBLI Standardized Blood Pressure Tables ^95^	X	X
Youth Mentor Outcomes				
Nutrition				
Food Attitudes and Preferences	Increase preference for fruits and vegetables	Fruit and Vegetable Preferences *Domel 1993* ^85^	X	X
Dietary Intake	Increase quality of diet (increase fruit and vegetable intake (quantity and variety), decrease intake of foods high in solid fats and added sugars; decrease sugar-sweetened beverages)	Youth Mentor reported 24-hour diet recall *Lindquist et al 2000* ^96^	X	X
Physical Activity				
Physical activity level and sedentary time	Increase estimated active time and decrease estimated sedentary time	SPAN questionnaire *Hoelscher et al 2010* ^89^	X	X
Mental Health				
Self-concept	Increase positive affect	PANAS Survey *Laurent et al 1999* ^90^	X	X
	Decrease negative affect			
	Retain positive changes in self-concept			
Social Support				
Social Support for Food and Physical Activity Habits	Increase social support for food and physical activity habits	Social Support Scale for Food and Physical Activity Habits *Gadhoke 2015* ^91^	X	X
Social Support for Healthy and Unhealthy Eating	Increase social support for healthy eating and decrease social support for unhealthy eating	Social Support Scale for Healthy and Unhealthy Eating *Fitzgerlad 2013* ^92^	X	X
Biometrics				
Height and Weight	Prevent unhealthy weight gain	Hopkins Road Rod Portable Stadiometer (Height) BalanceFrom High Accuracy Digital Scale (Weight) CDC sex-specific BMI-for-age growth chart^93^	X	X
Waist Circumference	Prevent increase in waist circumference	MyoTape tape measure CDC Waist Circumference Tables 94	X	X
Blood Pressure	Prevent increase in blood pressure	Panasonic Portable Blood Pressure Monitor NHBLI Standardized Blood Pressure Tables 95	X	X
Caregiver Outcomes				
Nutrition				
Caregiver Self-Efficacy	Improve caregiver self-efficacy to make healthy choices	Self-efficacy to make healthy choices *Bohman 2014* ^97^	X	X
Dietary Intake; Home Food Environment	Increase purchase and consumption of healthy foods; decrease purchase and consumption of unhealthy foods	Home Food Inventory *Fulkerson 2008* ^98^	X	X
Physical Activity				
Leisure Time Exercise	Increase engagement in leisure time exercise	Leisure Time Exercise Questionnaire *Godin & Shepherd 1985* ^99^	X	X
	Retain positive changes in leisure time exercise			
Anthropometrics				
Height and Weight	Prevent unhealthy weight gain	Self-report	X	X
Weight	Prevent unhealthy weight gain	Self-report	X	X

b0 = baseline; beginning of summer

t1 = post-intervention; end of summer

Interviews will be conducted at the home of the participants, the site, or another community location and are estimated to take approximately 30 min, 10 min, and 20 min to complete for the child, caregiver, and youth mentor, respectively. All assessment forms will be data collector-administered, where the data collector will read each question from the assessment form verbatim including all possible responses and record the participants’ response. If the participant provides an ambiguous response, the data collector will ask necessary questions to probe for a specific response. For younger children, caregivers will be asked to assist in completing and verifying responses from the child interview when deemed necessary.

#### Data analysis

The intervention will be tested by comparing change from baseline to post-intervention in diet quality, physical activity, mental health, and anthropometric outcomes for child participants (hypothesis 1.1) and psychosocial, physical activity, and anthropometric outcomes for adults (hypothesis 2.1). For each outcome variable of interest (Table 2), a mixed effects linear regression model will be fitted with site-type as the primary predictor. Other covariates will include race/ethnicity, income, and attendance, as well as baseline zBMI for all models that do not include weight status as the primary outcome. Using a mixed effects linear regression model allows us to capture the contributions of two sources of variability: (1) a between-site variability and (2) a between-subject or within-site variability. Impact of Camp NERF on Youth Mentors will be tested by comparing change from baseline to post-intervention in diet quality, physical activity, mental health, and anthropometric outcomes (hypothesis 3.1) using multiple linear regression analyses.

#### Process evaluation and environmental assessment

A Camp NERF Site Environmental Assessment Form was developed for this study and will be completed at baseline, mid-intervention (4 weeks), and post-intervention. The purpose of this form is to assess the demographic (i.e. predominant race/ethnicity of the staff at the sites), food environment (i.e. presence of vending machines, concession stands, and healthfulness of available foods), and physical activity environment (i.e. access to a gym, outdoor playground, equipment, etc.) characteristics of the sites. This information will be used in post-hoc analyses to determine if characteristics (i.e., access to a computer room) may have contributed to outcomes.

A Camp NERF Daily Process Evaluation Form was developed for this study and will be completed by trained process evaluators, who will not be involved with intervention implementation. This form assesses feasibility, fidelity, and acceptability of the intervention programming, assessment of food served, adherence to the USDA SFSP menus, and participant attendance.

### Intervention

#### Educator training and structure

The Camp NERF counselors will be undergraduate and graduate students in fields related to nutrition, kinesiology, public health, and education. The Camp NERF counselors will complete a 3-day, 24-h training prior to the launch of Camp NERF. The training will begin with an overview of our community partners, the USDA child meal programs, other pertinent issues, e.g., underutilization of the USDA SFSP and unhealthy weight gain during the summer months. Camp NERF Counselors also will be provided with a didactic overview of each of the core components of the Camp NERF program – nutrition, physical activity, and mental health – and will be given an opportunity to practice delivery of these curricula. Additionally, they will undergo training on topics related to education delivery and necessary for work with underserved children, as well as other components of the Camp NERF intervention. Table 3 provides an overview of the Camp NERF training for the Camp Counselors. In addition to the intensive training prior to intervention launch, the Camp NERF Counselors will attend a weekly staff meeting throughout the summer to provide feedback on the lessons from the current week, to coach the staff on improvement of curricula delivery and to practice lessons for the upcoming week.

**Table 3:** Camp NERF counselor training overview

Day 1	Welcome and Introductions
	Community Partner Overview
	Overview of the USDA SFSP
	“The Problem” and Our Place at the Table
	How to Keep Kids’ Attention
	How to Identify and Report Child Abuse and Neglect
Day 2	CATCH Nutrition Education Overview
	Practice CATCH Nutrition Education
	4-H Programming Overview
	Practice 4-H Programming
	COPE Education Overview
	Practice COPE Education
Day 3	CATCH Physical Education Overview
	Practice CATCH Physical Education
	Caregiver Engagement Overview
	Behavioral Economics Strategies Overview
	Meet and Greet with Youth Mentors

Three Camp NERF Counselors will be assigned to each site: One Counselor will serve as the kindergarten through second grade educator, one as the third through fifth grade educator, and one as the process evaluator. The Camp NERF Counselors at the Enhanced Care sites will be assisted by high school-aged adolescents - Camp NERF Youth Mentors - from the neighborhoods in which Camp NERF will be delivered.

#### Youth mentor-assisted education

The use of peer-led interventions have been utilized among youth in areas pertaining to the use of alcohol, tobacco, illegal drugs, violence, and sexual behavior [43–50]. Data indicate that mentored youth compared to un-mentored youth are less likely to participate in these aforementioned risky behaviors [51, 52] and are more likely to succeed academically [53–56]. Until recent years, use of peers as an intervention strategy to improve nutrition and physical activity, and ultimately weight status, had not been employed, but emerging research has demonstrated positive results for biometric-, nutrition-, and physical activity-related outcomes [43, 56–68]. According to the SCT, self-efficacy is influenced by role modeling the behavior [69]. As such, the peers leading the education may experience positive behavior change as a result of child mentoring. Unfortunately, the educating of peer mentors has been understudied [56, 64, 65, 67, 68].

Youth Mentors from the neighborhoods in which Camp NERF will be implemented will be recruited and will assist in the Camp NERF education delivery at the Enhanced Care sites. The Youth Mentors will undergo a 20-h work-readiness training, as well as will attend the 2-hour weekly Camp NERF staff meetings, where feedback will be provided on the lessons for the current week and the upcoming weeks lessons will be reviewed and practiced. Additionally, professional development topics, such as how to interact with co-workers in the workplace, will be discussed in collaboration with the undergraduate- and graduate-level Camp NERF Counselors.

#### Child education

##### Nutrition and physical activity

The Coordinated Approaches to Child Health (CATCH) Kid’s Club Healthy Habits and Nutrition Grades K-2 and Grades 3–5 curriculum and CATCH Kid’s Club Physical Education will be used for the Camp NERF nutrition and physical education components. The original CATCH program was initially implemented and evaluated from 1991–1994 in grades three through five in 96 schools in San Diego, CA, New Orleans, LA, Minneapolis, MN, and Austin, TX. Several positive findings on improvements in eating and physical activity behaviors came from these studies, including increased vigorous physical activity, decreased consumption of dietary fat, and reductions in children at-risk for being overweight and in children being overweight [70–74]. Due to the success of the original trial, the CATCH curriculum has continued to be adapted for and studied in various settings [75–82]. CATCH Kid’s Club is the modified curriculum for the after-school setting and has been shown to be effective in improving nutrition and physical activity knowledge and behaviors and reducing overweight and obesity.

##### Mental Health

The COPE curriculum will be the mental health component of the Camp NERF curriculum. COPE focuses on the thinking, feeling, behaving triangle and incorporates cognitive behavioral skill building in goal-setting, problem solving, coping, and emotional regulation [25]. The curriculum, originally developed for adolescents and young adults and more recently adapted to the younger audience of school-age children, consists of an introductory session and seven subsequent lessons. The lessons will be introduced and taught on the first day of Camp NERF each week, and the skills practice and review of lesson will be completed on the second day of Camp NERF each week.

##### 4H Programming

In order to assess whether potential differences demonstrated between participants is due to the type of programming delivered, as opposed to mere exposure to daily structured programming, an active control group was chosen for Camp NERF. Thus, the Camp NERF research team worked closely with 4-H Extension Specialists to identify non-nutrition and physical activity related programming suited for our target population. Sixteen lessons from the Cloverbud [83] curriculum will be delivered to participants at the Active Control sites.

#### Caregiver engagement

The caregiver engagement component will be in the form of a texting program that will utilize a mass messaging platform, social media (Facebook and Instagram), and traditional education materials. Adult caregivers of Camp NERF participants in the Enhanced Care group will be provided the option to receive three text messages each week over the course of the intervention. The first message each week will introduce the main nutrition topic that was presented to their child during programming but will encourage completion of a specific family nutrition goal to be attained by the end of the week. For example, the message preceding the fourth week of programming may read as follows: “Today at Camp NERF, your child learned about healthy fast food items. Are you in for trying healthier items at fast food restaurants? Please reply with ‘Yes’ or ‘No’.” The second message content will consist of either a strategy to assist the caregivers in reaching the weekly goal or educational information related to the topic of the week. The final message will inquire about achievement of the goal-setting challenge proposed at the start of the week.

Social media outlets such as Facebook, Twitter, and Instagram will be offered as an alternative means for caregivers to receive insight on the nutrition topic for the week. Images or videos will be added to the websites for caregivers to view and interact with counselors as well as other caregivers. Nutrition topics will be explored in greater depth, such as links to simple food recipes, news items, and recent educational articles related to the weekly topic.

In addition to the text-messaging and social media campaigns, traditional educational materials also will be disseminated to caregivers at the Enhanced Care sites weekly. These educational materials are adapted from the CATCH Kids Club Healthy Habits and Nutrition curriculum handouts for parents [26]. The handouts are modified to include concepts from the CATCH Physical Education curriculum and the COPE curriculum [26, 27]. Child participants will be given handouts at the end of the week to take home to their caregivers.

## Discussion

Despite the recent plateauing in prevalence, the number of obese children remains high, which is problematic due to the negative, short- and long-term health consequences for children [1–3]. Emerging research has indicated the summertime as a particular window of risk for unhealthy weight gain among children, especially underserved, minority children [4–10]. Few efforts have been directed at designing evidence-based nutrition and physical activity programs to equip children with the necessary knowledge, skills, and other resources to prevent excess weight gain during the summer recess.

The purpose of this paper is to describe the aims and research methods of Camp NERF, a multi-component, evidence-based nutrition, physical activity, and mental health intervention coupled with USDA SFSP open sites in Columbus, Ohio to address the disproportionate childhood weight gain in underserved children during the summer months. To our knowledge, Camp NERF is the first evidenced-based nutrition education research study and program to address this issue. This study will fill a critical research void and provide insight for effective programming to address child health during the summer months. The Camp NERF program is coupled with the USDA SFSP and utilized existing systems for implementation, which ensures the future sustainability of the program.

Several challenges or limitations have been identified. Engagement from caregivers in the target population may be a challenge, as it is a common issue with intervention research involving underserved families [84, 85]. However, this study was developed and designed to overcome this barrier. The research team and community collaborators will be present in the participating neighborhoods for several years throughout the development of the project. High-school aged students from the intervention communities will be engaged as Youth Mentors and will assist with education delivery throughout the entirety of the program. Traditional (educational handout materials) and innovative (text messaging and social media) strategies will be utilized to inform parents about the programming and encourage participation. Another limitation is the lack of a true negative control group. Because Camp NERF will be coupled with the USDA SFSP, a federal child nutrition program, the statutory right for participation applies. Ethically, the research team cannot ask children and families to not participate at the open SFSP sites. Additionally, recruitment methods are not designed to seek participants who do not intend to attend the SFSP sites during the summer.

In summary, Camp NERF builds on successful childhood obesity prevention interventions which include nutrition and physical activity components, concurrent knowledge and skill building, coupling of the intervention curriculum to availability of healthy foods, and opportunity for physical activity and play [18, 86]. Studying the impact of such an intervention over the summer months will provide valuable information in tackling a time period during which children may be at increased risk for excessive weight gain.
